# Osteoid Osteoma Treated with Radiofrequency Ablation

**DOI:** 10.1155/2015/807274

**Published:** 2015-02-02

**Authors:** Murat Çakar, Cem Zeki Esenyel, Metin Seyran, Ali Çağrı Tekin, Müjdat Adaş, Mehmet Kürşad Bayraktar, Ünsal Coşkun

**Affiliations:** ^1^Orthopaedics and Traumatology Clinic, Okmeydanı Training and Education Hospital, Şişli, 34834 Istanbul, Turkey; ^2^Radiology Clinic, Okmeydanı Training and Education Hospital, Şişli, 34834 Istanbul, Turkey

## Abstract

*Purpose.* Our aim is to evaluate the results of treatment with computed tomography (CT) guided percutaneous radiofrequency ablation for osteoid osteomas which were localized in a difficult area for operation. *Materials and Methods.* Glenoid, distal tibia, humerus shaft, proximal humerus, and in third finger of the hand proximal phalanx were involved in one patient. Proximal femur was involved in three patients, distal femur was involved in three patients, and proximal tibia was involved in two patients. 9 males and 4 females were aged 4 to 34 years (mean age: 18.5 years). All patients had pain and were evaluated with X-rays, CT, bone scintigraphy, and MRI. In all patients, RF ablation was performed with local anesthesia. The lesion heated to 90°C for 6 minutes. *Results.* All of the patients achieved complete pain relief after ablation and were fully weight bearing without any support. In all patients, there was soft tissue edema after the procedure. During follow-up, all patients were free from the pain and there was no sign about the tumor. There was no other complication after the process. *Conclusion.* CT guided RFA is a minimally invasive, safe, and cost-effective treatment for osteoid osteoma placed in difficult area for surgery.

## 1. Introduction

Osteoid osteoma, first described by Jaffe in 1935, is a benign osteoblastic lesion characterized by a nidus of osteoid tissue, constituting 10% of all benign bone tumors [1, 2]. Osteoid osteoma occurs in the young, usually between the ages of 10–35 with a male predominance [3, 4]. In over 50% of cases, they are centered on the cortex of the diaphysis of the femur or tibia with the proximal femur being most frequently affected [5]. Other common sites include the spine, hands, and feet. The most important clinical symptom is pain that is more severe at night and responds to salicylates or other nonsteroidal anti-inflammatory drugs well [2]. This is due to the secretion of prostaglandins in varying degrees [6, 7]. Other possible symptoms include growth disturbances, bony deformity, and painful scoliosis, and if located within the capsule of a joint, they include swelling, synovitis, restricted movement, and contracture [8]. Osteoid osteoma was diagnosed clinically and radiologically. Radiographs characteristically show a circular or ovoid lucency representing the nidus (usually less than 1.5 cm in diameter) with a variable degree of surrounding sclerosis [9]. Radionuclide bone scanning is sensitive but has low specificity; computed tomography (CT) is more effective than magnetic resonance imaging for making diagnosis and localizing the tumors [10].

There are different treatment options for osteoid osteoma including surgical, conservative, and percutaneous techniques [11]. The purpose of this study is to report our experience of the technique and clinical problems in patients with osteoid osteomas treated with CT guided radiofrequency ablation.

## 2. Materials and Methods

Between June 2010 and October 2011, 13 patients (9 males and 4 females) with osteoid osteoma were treated with percutaneous CT guided radiofrequency ablation technique. The mean age at presentation was 18.5 years (range 4–34 years). The diagnosis is based on severe pain, relief of pain after administration of NSAID, and radiological features. The duration of pain before application to the outpatients department varied from 3 months to 42 months. All patients had received medical therapy with NSAID before procedure. We confirmed the nidus smaller than 1.5 cm in diameter on radiography, computed tomography, and magnetic resonance imaging. The median nidus size was 6.8 mm (range 3.4–1.4 mm). No patients had previously undergone surgery for the lesions. Lesions were located in proximal femur (*n*: 3), distal femur (*n*: 3), distal tibia (*n*: 1), proximal tibia (*n*: 2), humerus shaft (*n*: 1), proximal humerus (*n*: 1), scapula (*n*: 1), and phalanx (*n*: 1).

Patients were informed of alternative treatments and informed consent was obtained from the patients or their parents.

### 2.1. Technique

**Figure 1:**
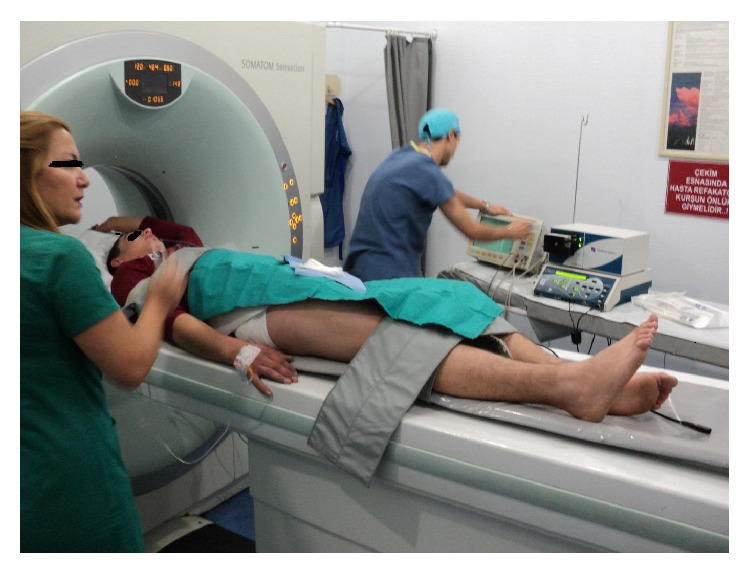
The patient was positioned on CT bed.

**Figure 2:**
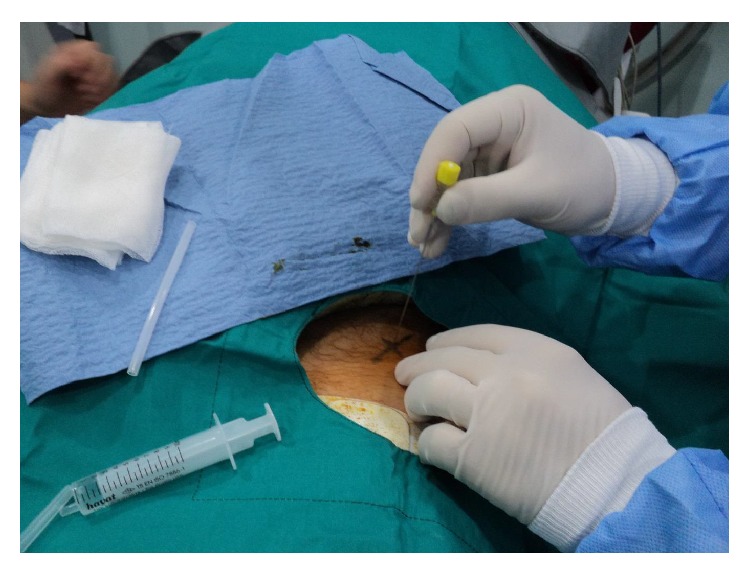
Entry point is marked.

**Figure 3:**
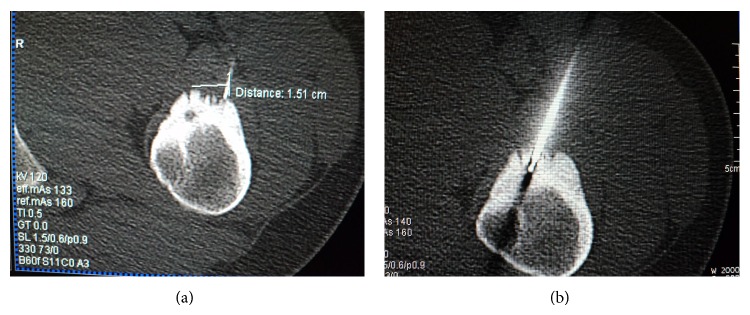
Spot CT images are obtained to confirm appropriate positioning.

**Figure 4:**
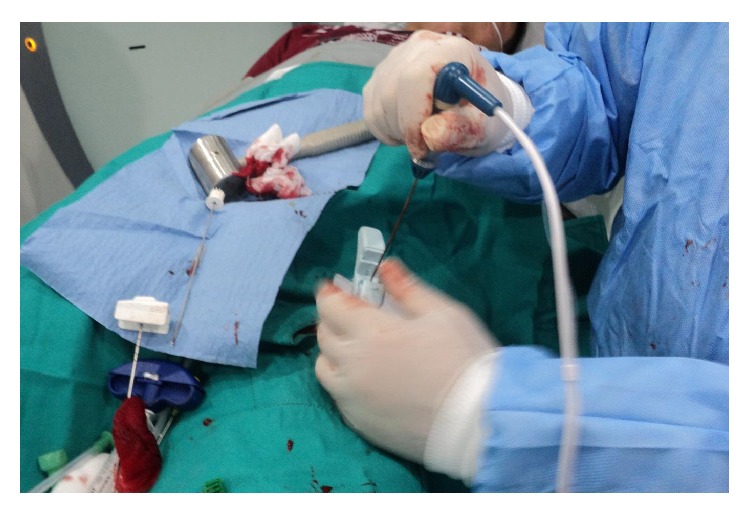
The trochar removed and electrode was placed through cannula.

**Figure 5:**
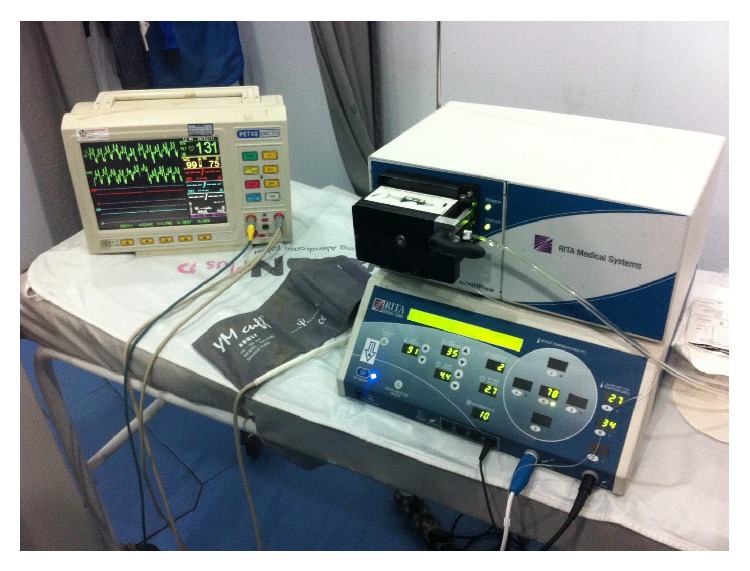
The electrode was connected to the RF generator and thermal ablation is applied.

All procedures were performed in CT room under aseptic conditions with local anesthesia. The patient was positioned on CT bed and the lesion localization was confirmed by CT imaging (Figure 1). The preferred approach is at angle perpendicular to the cortical surface of the bone (because oblique approach to the bone surface could risk the needle skidding) and shortest distance through the bone (if there is a neurovascular or other anatomic structures, the entry must be through the opposite normal cortex of bone). Entry point is marked and a small skin incision was made at the entry point and blunt dissection was done (Figure 2). A trochar and cannula were advanced to the bone and the trochar was tapped until it advanced into the nidus. Spot CT images are obtained to confirm appropriate positioning (Figures 3(a) and 3(b)). The trochar was removed and electrode (UniBlate, AngioDynamics, Inc., USA) was placed through cannula (Figure 4). The electrode was connected to the RF generator (RITA 1500X, AngioDynamics, Inc., USA) and temperature is increased from 77°C to 90°C in 2-3 minutes (Figure 5). Thermal ablation is applied with RF electrode at 90°C. Ablation is done for a total of 4–6 minutes. After ablation, the electrode is removed, a local anesthetic is injected for pain relief, and a compressive sterile dressing is applied. All daily activities are resumed immediately after the procedure. All patients were discharged from hospital on the same day unless the pain was too severe. A follow-up visit is scheduled for one month and six months after the procedure.

## 3. Results

The needle was placed within the nidus in all patients. Each nidus was reached with the RF electrode at the first attempt.

To access the technical success, each patient was examined before discharge and evaluated for bleeding, swelling, burn, neurovascular complications, and other procedure related problems. Electrode was placed in the nidus and the procedure was technically successful in all cases and there was no procedure related complication. To access the clinical success, each patient was examined one month and six months after procedure and evaluated with X-rays, MRI, and a questionnaire. If there was pain relief, no increase in the symptoms and no recurrence in radiologically the procedure classified as successful. In all cases, the procedure was successful. All of the patients achieved complete pain relief within the first week after radiofrequency ablation. All patients were fully weight bearing without any support. There was no activity restriction in all patients after the procedure. In all patients, there was soft tissue edema which is seen on MRI up to three weeks after the procedure. There was no clinical reflection of this radiologic sign. The mean follow-up time was 5.3 months. No recurrence occurred during the follow-up period.

## 4. Discussion

There are different treatment options for osteoid osteoma including surgical, conservative, and percutaneous techniques [11]. Osteoid osteomas can be treated conservatively with nonsteroidal anti-inflammatory drugs because osteoid osteomas may undergo spontaneous regression after several years [12, 13]. Complete surgical excision is the classic treatment if the conservative treatment fails and the pain persists [10, 14]. But this method may result in the wide resection of normal bone to ensure completely excise of the tumor [14]. This causes structural weakening and requires a long period of limited weight bearing and activity restriction [15, 16]. Surgery in anatomically difficult sites such as acetabulum, glenoid, and femoral head or neck also carries high risk of complications [10, 17]. The clinical success rate of surgery ranges from 88 to 100% [18]. Disadvantages of open surgery have made percutaneous techniques as an alternative. Percutaneous techniques may be divided in two groups: those that attempt to remove the lesion physically and those that aim at in situ ablation [19]. In the first group, the procedure is percutaneous excision with large-caliber hollow needles and drills. Ablative techniques include ethanol injection, laser photocoagulation, and radiofrequency treatment. Radiofrequency ablation (RFA) involves the use of thermal coagulation of the nidus to induce necrosis in the osteoid osteoma. RFA and open surgical treatment have equivalent treatment outcome [20]. RFA was preferred because of shorter hospital stay and shorter recovery time. Percutaneous RFA was applied as a day surgery in our clinics. Clinical success with these methods varies between 70 and 100% [21]. In our study, the clinical success was 100%. We acknowledge some disadvantages in our study. First is the limited number of patients. Second is the lack of histological verification of the osteoid osteoma. We considered typical clinical symptoms and radiological findings sufficient to make diagnosis as other authors [8, 22]. Third is the short follow-up period.

Various needle guidance approaches have been described as minimally invasive and effective methods for the percutaneous treatment of the osteoid osteomas, such as computed tomographic (CT) guidance [8, 11, 23, 24], fluoroscopic guidance [25], ultrasonographic guidance [26], or magnetic resonance (MR) guidance [27]. Ultrasonographically, osteoid osteoma is difficult to distinguish. So therefore it is not true modality of choice [28, 29]. Recently MR guided laser ablation techniques related articles are available but CT guided RFA has advantages. These advantages include the highly resolved visualization of bone structures and rapid frame rate, allowing treatment under real-time fluoroscopy [27].

Computed tomography is a good technique to visualize the nidus and its surrounding soft tissue. However, it has some limitations in needle guidance. Real-time guidance is not possible. Because of that, CT-fluoroscopy is used. We used CT-fluoroscopy to place the needle correctly into the nidus. All patients in the study are free of the nidus during the follow-ups.

Previous studies reported complications such as skin burn, skin and fat necrosis, soft tissue infection, vasomotor instability, tendinitis, and hematoma [8, 10, 11, 14, 30, 31]. In our study, there was no complication. In all patients, there was soft tissue edema after the procedure and there was no clinical reflection of this radiologic sign. It relieved in 3 weeks' time. In English literature, this was not reported before. We believe that this is a result of prostaglandin discharge from osteoid osteoma during ablation. But we do not have sufficient histological studies to provide this. In our study, RF ablation is performed with local anesthesia. Some authors prefer general anesthesia for adequate pain control and stable position during the ablation [10, 11]. We did not have any difficulty to maintain adequate pain control and position stabilization with local anesthesia. In addition, we did not have any anesthesia related complication.

RF ablation for treating osteoid osteoma was first described in 1989 [32], with initial results published in 1992 [23]. The procedure is minimally invasive, safe, and effective and has many advantages for the treatment of osteoid osteoma. For these reasons, RFA should be considered the current method of choice for treatment [9].
